# Exploring Tumor Heterogeneity Using PET Imaging: The Big Picture

**DOI:** 10.3390/cancers11091282

**Published:** 2019-08-31

**Authors:** Clément Bailly, Caroline Bodet-Milin, Mickaël Bourgeois, Sébastien Gouard, Catherine Ansquer, Matthieu Barbaud, Jean-Charles Sébille, Michel Chérel, Françoise Kraeber-Bodéré, Thomas Carlier

**Affiliations:** 1 CRCINA, INSERM, CNRS, Université d’Angers, Université de Nantes, 44093 Nantes, France; 2 Nuclear Medicine Department, University Hospital, 44093 Nantes, France; 3 Groupement d’Intérêt Public Arronax, 44800 Saint-Herblain, France; 4 Nuclear Medicine Department, ICO-René Gauducheau Cancer Center, 44800 Saint-Herblain, France

**Keywords:** PET, heterogeneity, radiomics, radiopharmaceuticals, SUV, nuclear medicine

## Abstract

Personalized medicine represents a major goal in oncology. It has its underpinning in the identification of biomarkers with diagnostic, prognostic, or predictive values. Nowadays, the concept of biomarker no longer necessarily corresponds to biological characteristics measured ex vivo but includes complex physiological characteristics acquired by different technologies. Positron-emission-tomography (PET) imaging is an integral part of this approach by enabling the fine characterization of tumor heterogeneity in vivo in a non-invasive way. It can effectively be assessed by exploring the heterogeneous distribution and uptake of a tracer such as 18F-fluoro-deoxyglucose (FDG) or by using multiple radiopharmaceuticals, each providing different information. These two approaches represent two avenues of development for the research of new biomarkers in oncology. In this article, we review the existing evidence that the measurement of tumor heterogeneity with PET imaging provide essential information in clinical practice for treatment decision-making strategy, to better select patients with poor prognosis for more intensive therapy or those eligible for targeted therapy.

## 1. Introduction

Heterogeneity is a concept familiar to pathologists. Phenotypical and functional differences arise among cancer cells during the course of the disease because of genetic changes [1]. Similarly, the interactions of cancer cells with their microenvironment or the local variation in angiogenesis and hypoxia are not uniform in the tumor. Not to mention the perpetual clonal remodeling under the pressure of microenvironment and treatments. This large biological, cellular, and tissue heterogeneity exist at the intratumoral level (molecular differences within one tumor), intrapatient level (variation of tumor features between lesions within one patient), and interpatient level (variation of tumor features between patients). This heterogeneity conditions tumor aggressiveness and therapeutic resistance and represents a significant challenge in the design of effective treatment strategies [2]. The prerequisite for personalized medicine relies on the report of such heterogeneities. Yet, the realization of multi-region sampling from each tumor of a single patient raises ethical or technical questions. Positron-emission-tomography (PET) imaging appears as a perfect tool to overcome this obstacle, providing a whole-body non-invasive method of assessing tumor heterogeneity, through the use of multiple radiopharmaceuticals, each providing different information. In parallel, the information derived from the uptakes’ analysis of a tracer such as 18F-fluoro-deoxyglucose (FDG) has enabled the emergence of a wide variety of PET quantitative metrics including simple semi-quantitative approaches such as standardized uptake value (SUV) and “high-order metrics” that involve a segmentation step and supplementary image processing. These parameters, besides their utility for therapeutic response, should play a key role in the prognostic characterization of tumors, along with the development of personalized medicine. Radiomics—the high throughput extraction of large amounts of imaging elements from radiographic images—tackles this challenge and is one of the most promising strategies [3,4]. The purpose of this short review is to present the latest developments in the exploration of tumor heterogeneity in PET imaging. Different examples of neoplasias are presented during the three developed axes. Nevertheless, to emphasize to the reader that these tools can be used in all diseases, lymphoma is used as a common thread throughout this review.

## 2. Inter- and Intra-patient Tumor Heterogeneity Exploration through Multiple Tracers PET Imaging

Nuclear medicine is one of the most dynamic medical fields, in constant evolution over the past decades. The main strength of this discipline lies in its incredible catalog of radiopharmaceuticals allowing exploration of virtually every major organ system in the body (Table 1). Personalized medicine has never been so relevant today and nuclear medicine is on its leading edge, probing deep inside each patient or tumor to reveal its inner workings. Predictive biomarkers are an essential tool of precision medicine and individualized treatment. Yet, as mentioned above, tumor heterogeneity contributes to sampling error, especially for metastatic diseases; target’s expression at one site does not guarantee expression at all sites. Moreover, target accessibility of drugs is not assessed by biopsy, and target expression does not provide evidence of targeted-therapy impact on the target. In this context, PET imaging overcomes many of these limitations exploring target heterogeneity, assessing target expression and potential accessibility across the whole disease burden, to aid clinical decision making. A perfect example was recently published by Bensch et al. with the initial results from the first-in-human imaging with 89Zirconium-labeled atezolizumab [5]. The programmed cell death protein 1 (PD1)/programmed death-ligand 1 (PD-L1) axis is an important immune checkpoint for T-cell activation. PD-L1 overexpression is associated with a poor prognosis in a variety of cancers yet these patients typically have a stronger response to anti-PD-L1 therapy such as atezolizumab [6,7,8,9]. The PD-L1 expression is usually evaluated using immunohistochemistry or RNA sequencing. In Bensch et al. study, clinical responses were better correlated with PET uptake before treatment than these two evaluations.

Breast cancer also represents a great model for this type of approach [12,13]. Indeed, in this pathology, 18F-fluoroestradiol (FES) PET has been validated as an accurate method for providing information on estrogen receptor (ER) status of tumor lesions to determine need for endocrine therapy [14,15,16,17]. Indeed, the uptake of FES has been proven to correlate with ER expression in biopsy sample [16]. Similarly, several works showed that radiolabeled monoclonal antibodies could non-invasively identify lesions with positive or over-expression of the human epidermal growth factor receptor 2 (HER2) and predict response to anti-HER2 antibody-based therapy [18,19,20,21]. In particular, Gebhart et al. recently reported the promising results of the ZEPHIR trial. This work successfully evaluated two PET imaging as tools to investigate heterogeneity of advanced HER2-positive breast cancer (PET imaging using trastuzumab radiolabeled with 89Zirconium) and to predict patient outcome under trastuzumab emtansine (PET imaging with FDG) [22,23]. This innovative study showed the clear benefit of combining both imaging methods in predicting whether adequate tumor targeting is followed by sufficient efficacy and cytotoxicity.

**Figure 1:**
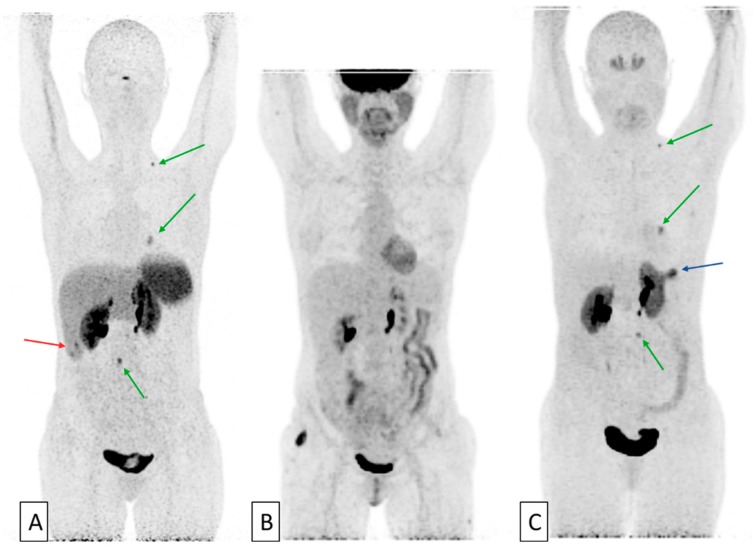
An example of a 46-year-old patient with pluri-metastatic intestinal neuroendocrine tumor (grade 2, ki67 at 4%). 68Ga DOTA-TOC (**A**), FDG-PET (18F-fluoro-deoxyglucose- positron-emission-tomography) (**B**) and FDOPA (18F-fluorodihydroxyphenylalanine) (**C**) imaging were realized. MIP (maximum intensity projections) images from the respective PET data sets are shown. The subject has positive on both FDOPA and somatostatine-receptor imaging, dominant disease which exhibits no FDG uptake (green arrows). One hepatic lesion was FDOPA-negative and 68Ga-DOTA-TOC-positive (red arrow) and one gastric lesion was FDOPA-positive and 68Ga-DOTA-TOC negative (blue arrow). Images courtesy of Pr C. Bodet-Milin.

**Figure 2:**
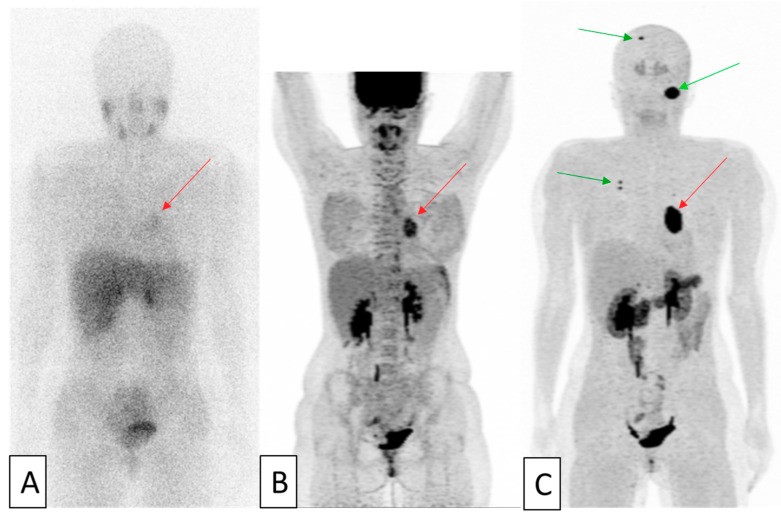
An example of 37-year-old patient with pluri-metastatic paraganglioma. MIP (maximum intensity projections) images of the realized 123MIBG-scintigraphy (**A**), FDG-PET (**B**) and FDOPA-PET (**C**) are shown. The subject has a mediastinal lesion, barely seen on 123MIBG-scintigraphy and clearly positive with the others tracers (red arrows). Pulmonary and skull lesions (green arrows) were only visible on FDOPA-PET. Images courtesy of Dr C. Ansquer © Catherine Ansquer

Beyond the “simple” search for the expression of a target before initiating a treatment directed against it, the use of several radiotracers in the same patient can allow to comprehensively assess disease activity, extent, and heterogeneity. Tumors derived from cells of the neural crest represent the perfect historical model for this approach [24]. This large group of neoplasms includes a large variety of tumors, such as gastroenteropancreatic neuroendocrine tumors (GEPs), neuroblastoma, paraganglioma, pheochromocytoma, medullary thyroid carcinoma, and small cell lung cancer. These tumors are characterized by similar appearances and expression of different peptides and amines. Prognostic criteria of this group are generally related to the metastatic extension of the disease but also to the functional activity, degree of differentiation of the tumor and proliferative indices. These parameters are essential in the management of these patients. Today, multiple molecular imaging methods are available to explore these various biologic and histologic characteristics with very high specificity and can be performed at the whole-body scale [25,26]. Tracers used can be grouped in three different categories. 123-metaiodobenzylguanidine (123MIBG), an analog of norepinephrine and 18F-fluorodihydroxyphenylalanine (FDOPA), an amine precursor, exploit catecholamine synthesis, storage, and secretion pathways. 111In-pentetreotide and 68Gallium-labeled somatostatin analog peptides (68Ga-DOTA-TOC, 68Ga-DOTA-NOC, 68Ga-DOTA-TATE) assess the somatostatin receptors expression. Finally, FDG uptake has been found to correlate with de-differentiation, increasing aggressiveness and proliferation rate, and poor prognosis. This phenomenon was first described in differentiated thyroid carcinomas. Indeed, de-differentiated thyroid carcinomas lose their capacity to capture radioiodine and can be detected by FDG-PET since glycolysis increase at the same time. Thus, imaging assessment of two or more tracers may yield more clinical information than each alone [27,28,29,30,31,32] (Figure 1 and Figure 2).

Lymphomas represent another cancer group where multiple tracers’ exploration might allow a better potential characterization of tumor heterogeneity. Indeed, today, FDG-PET occupies a central position in accurate staging and therapeutic evaluation of lymphomas [33]. Nevertheless, a certain number of crucial questions remain regarding its optimal application. While risk-based strategies may appear to enhance patients’ outcomes for those with Hodgkin’s lymphoma, findings are not so impressive in non-Hodgkin’s lymphoma [33]. Similarly, novel therapies that may generate an immune response may lead to false-positive FDG-PET results, requiring the incorporation of these flare-ups reactions in existing interpretation criteria [34]. Therefore, the development of other radiotracers with different uptake mechanisms from FDG could be of interest [35]. 18F-fluorothymidine (FLT) and 11C-methionine (MET), for instance, were both reported to correlate with cellular proliferation activity and lymphoma histological grade of malignancy, respectively, through the exploration of DNA and protein synthesis [36,37]. Moreover, FLT showed excellent results in treatment monitoring and particularly in the setting of early interim evaluation, with more specific and accurate analyses than FDG [38,39]. In the same way, 18F-fludarabine, an adenine nucleoside analog, owing to its specificity for lymphoid cells and its absence of uptake in inflammatory tissues, holds great promise for therapeutic evaluation [40]. Finally, the first in-human study of 68Ga-CXCR4, targeting chemokine receptor CXCR4, which is frequently overexpressed in various tumor types, showed high lesions’ uptake [41,42]. Moreover, voxel-by-voxel analysis in one patient identified striking inter- and intralesional heterogeneity in the uptake of 68Ga CXCR4 and FDG, implying that the biological information given by the two probes may be complementary even in lesions that show avidity for both [42].

Systematic multiple tracers imaging could be used to reveal different profiles with highly different prognoses. This multiple-tracers imaging associated with an appropriate scoring system might also influence patients’ management and help selecting between different therapy options [28,43,44]. Indeed, these tumors may be treated with molecular radiotherapy using the same pathways: 131-metaiodobenzylguanidine (131-MIBG) and 177Lutetium-DOTA-TATE. Impressive reports were reported with these targeted therapies in GEPs and neuroblastoma [45,46,47,48,49]. A theranostic approach, integrating imaging and therapy in the same system, providing individualized tailored treatment, despite intratumor and interlesional heterogeneities, is expected to play an increasingly pivotal role in this large tumor group [50,51].

The theranostic approach indeed represents a formidable field of expansion for nuclear medicine in an era where targeted therapies have become essential tools in oncology pharmacopoeia [52,53]. As described above in breast cancer and neuroendocrine tumors, it offers a non-invasive method for quantitatively evaluating target expression in vivo, selecting patients for costly and potentially toxic treatments, and monitoring responses [50,51,54,55,56]. In addition, this approach constitutes a valuable asset in the development of new drugs by pharmaceutical companies. Drug development being a fairly time-consuming and costly process, it represents an effective solution to rapidly monitor drug candidates’ pharmacokinetics and biodistribution. This strategy can improve the strength and effectiveness of early trials by enhancing patient selection, optimizing dose, and rationalizing treatment reactions [54].

## 3. Intrapatient Tumor Heterogeneity Exploration through Quantitative Analysis of PET Imaging

FDG-PET has become an essential tool for cancer diagnosis, staging, and therapeutic evaluation. It has undoubtedly changed the landscape of lymphoma, lung, head and neck or breast cancers management [57,58,59]. This spread of the PET imaging technique was particularly enabled by its quantification ability which allows the use of a reproducible metric for cancer monitoring. The SUV and particularly SUVmax (defined as the SUV value of the maximum intensity voxel within a region of interest) is widely used in everyday clinical practice. It is popularly adopted as a surrogate of tissue accumulation of tracers and particularly as the overall net rate of FDG uptake. It is defined as the ratio between the radiopharmaceutical concentration (expressed in Bq/mL) and the decay-corrected injected activity normalized by a given factor (mass of the patient, body surface area or lean body mass) [60]. The precise description of underlying technical limitations of this metric is beyond the scope of this short review and is already largely discussed in the literature [60,61,62]. Only its use under clinical situation is highlighted here. Indeed, the computation of intrapatient tumor heterogeneity in FDG-PET imaging using SUV has been already applied in a wide variety of indications even if not intentionally or explicitly. FDG uptake heterogeneity may reflect different tumor profiles with different aggressiveness and consequently prognosis. This contribution offered by the calculation of SUV has been particularly investigated in lymphoma.

Some histological subtypes of lymphoma such as follicular and mantle cell lymphomas are heterogeneous diseases with a variety of clinical, genetic, biological features and related different outcomes. In these pathologies, the intensity of FDG uptake on PET imaging at baseline varies greatly between patients [33]. Some present barely detectable uptakes while others exhibit very intense fixations. Yet, this spectrum of SUV, from very low to very high uptakes, provides clinically relevant information. Indeed, several reports demonstrated that the level of FDG uptake on PET imaging is largely correlated to lymphoma histology [63,64]. More particularly, indolent disease with low proliferation rate is generally associated with low uptake, while a more aggressive disease presented higher FDG uptake. In the same way, very intense FDG uptake associated to a clear uptake gradient may pertain transformation from indolent to aggressive lymphoma. These findings were confirmed in a prospective study conducted to evaluate the value of FDG-PET as an accurate guide for biopsies in suspected transformed tissues [65]. In patients with newly diagnosed indolent lymphoma, low SUV numbers may reduce the suspicion of transformation in disease sites that were not biopsied. Conversely, in patients with histologically proven indolent lymphoma, an uncharacteristically higher than expected SUV may herald an aggressive subtype, warranting a targeted biopsy. Our team reported similar findings in mantle cell lymphomas. A broad inter-individual tumor cell heterogeneity regarding FDG avidity was observed in several works with a strong prognostic value on survival of quantitative parameters such as SUVmax of the lesions with the highest uptake determined at diagnosis [66,67,68,69]. This close relationship between high SUVmax values and a more aggressive mantle cell lymphoma behavior was also supported by the concordance between SUVmax, aggressive variants, and high percentage (> 30%) of Ki67 positive cells.

**Figure 3:**
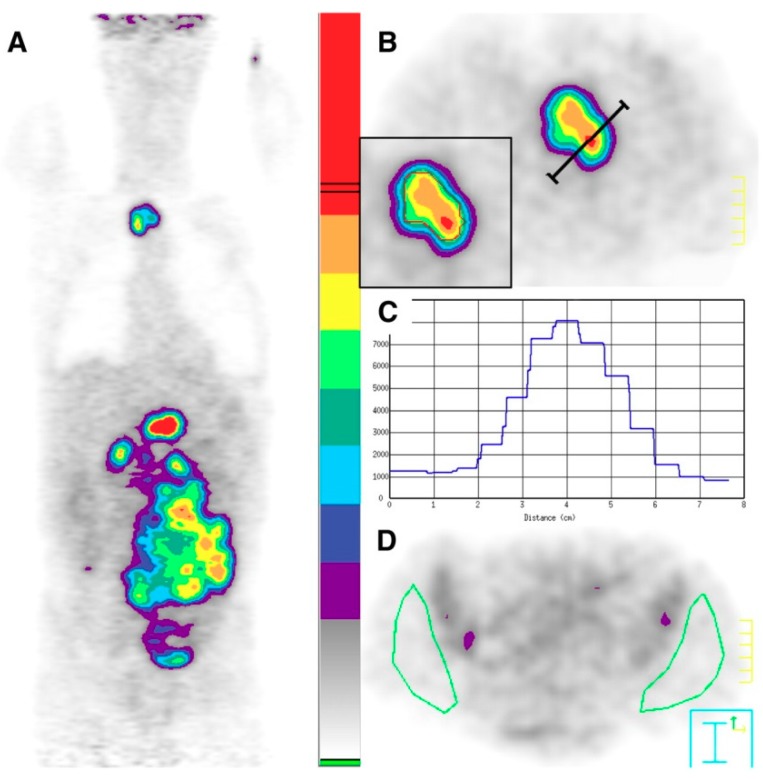
Selection of regions of interest in 57-y-old patient before chemotherapy. (**A**) Graded color-scaled parametric analysis applied in reconstructed coronal PET image shows most active tumor in upper abdomen. (**B**) Transverse PET image with a higher scale reveals celiac tumor (T) with activity profile crossing the hottest point (red spot). (**C**) Corresponding activity profile in counts-per-pixel. Isocontours are drawn with lower autocontour threshold of 4500 counts-per-pixel (red isocontour at inset in B). (**D**) Normal background tissue (N): two large ROIs are manually selected on gluteal muscles, avoiding iliac bone marrow activity. This research was originally published in JNM [76]. © SNMMI.

This hypothesis that the prognosis of the disease is linked to the most aggressive contingent corresponding to the lesion with the highest FDG uptake is also reinforced by some studies exploring the predictive prognostic value of FDG-PET during treatment. Since the development of normalized criteria for assessment of tumor burden changes, the required number of lesions to consider for response determination remained a fundamental question [70,71,72]. The RECIST criteria used for radiological evaluation recommended the measurement of five target-lesions, selected “randomly,” only on their suitability for accurate repeated measures [70]. On the contrary, several works using PET imaging showed that only the most metabolically active lesions, representing the most aggressive portions of tumors, are critical to consider [73,74,75,76,77]. This approach implies taking the single hottest area as the reference point on the pre-treatment and post-treatment studies, even if not necessarily the same area, considering only the worst biologic behaviors of the malignancy. This controversial concept was explored in many cancers and was applied both in solid tumors with the PERCIST criteria and in lymphomas. For example, Lin et al. were the first to measure the reduction of SUVmax in the “hottest” lesion before and during the treatment, in diffuse large B-cell lymphoma (Figure 3). In their study, they also investigated the FDG uptake changes on interim FDG-PET within the initial hottest tumor site on baseline FDG-PET (18% of 92 patients) which resulted in more false-negative exams in predicting PFS [76]. 

This approach was also recently reported in multiple myeloma patients [78]. In this pathology, multi-clonal heterogeneity remains one of the main challenges in developing effective strategies. Multiple myeloma is indeed characterized by spatial differences in the clonal architecture, with potential non-homogeneous distribution of high-risk disease for which multi-region investigations appear critical [79,80,81]. Yet, in a study by our team, the percentage difference of SUVmax between baseline and interim FDG-PET was a powerful tool to predict long-term outcomes in patients with FDG-avid multiple myeloma [78]. There again, similar to previous work in lymphomas or in solid tumors, the hottest lesion in any region was used for comparison even if its location differed from the initial hottest lesion on PET at diagnosis, to assess the most aggressive portion of the disease on each PET examination, to free oneself from intrapatient heterogeneity.

## 4. Intratumor Heterogeneity Exploration through Quantitative Analysis of PET Imaging

**Figure 4:**
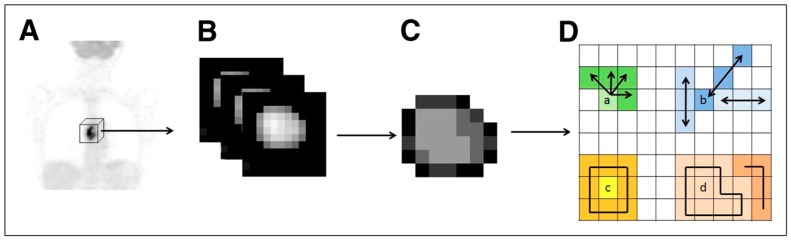
Whole-body 18F-FDG PET scan (**A**) tumor segmentation (**B**) and voxel-intensity resampling (**C**) allowing extraction of different features (**D**) by analysis of consecutive voxels in a direction (for cooccurrence matrices) (a), alignment of voxels with same intensity (b), difference between voxels and their neighbors (c), and zones of voxels with same intensity (d). This research was originally published in JNM [90]. © SNMMI.

In addition to conventional measurements of SUV, a new class of metrics has recently emerged in PET imaging and is currently being clinically investigated [61]. A simple visual analysis of the FDG uptake in PET images indeed suggests that the spatial distribution of voxels of different intensities in a selected region and thus the spatial distribution of the radiotracer can be extremely heterogeneous. And one can assume that this localized heterogeneity in medical images “partly” reflects heterogeneity on a lower scale and underlying variations in metabolism, cellular proliferation or necrosis [82]. Advanced image analysis of a tumor could then capture additional information and some researchers suggested that genomic, proteomics, and other -omics patterns could be expressed in terms of macroscopic image-based features [83]. This concept requiring the extraction of a large number of quantitative data from medical multimodal images has become popular under the term “radiomics” [3,84,85]. In recent years, considerable efforts have been made by the medical imaging community to obtain correlations between these image characteristics and tumor heterogeneity. Those metrics are often referred to as “textural features” and belong to “high order parameters” (Table 2) along with shape-descriptors or other descriptors based on fractal analysis or wavelet decomposition [61,86]. They measure the relationships between groups of two or more voxels in the image (Figure 4). Numerous textural features can be extracted from medical images, yet only a handful are sufficiently reliable, robust, and reproducible. Texture analysis remains limited and biased by many methodological and technical factors inherent to PET images’ acquisition, reconstruction algorithms, or segmentation technique that can affect the quantification of image heterogeneity [86]. A number of recommendations are available to help and guide researchers in making the right choices in the calculation and selection of parameters [86,87,88,89].

To date, numerous studies explored the potential value of textural features in PET imaging with encouraging results in a number of cancers [86,92]. However, only a limited number adopted rigorous methodological choices with particularly large cohorts of patients and robust statistical analysis [93,94]. Keeping in mind these limitations, the evidence supporting the additional value of advanced image features from FDG-PET continues to expand year after year. Several of the most recent studies have used techniques such as external cohort validation [95,96,97], or even machine-learning technique [98,99] and concluded in the usefulness of textural analysis regarding patient management. Several method were also proposed to minimize the effect of inter-center variability related to textural features computation [95,100,101] with encouraging results both on a methodological and prognostic level. One should keep in mind that several hundred, if not thousands of handcrafted features can be extracted, when the number of patients used to construct the predictive model is often several order of magnitude lower than the number of features analyzed. The use of machine learning approaches in this context is thus very useful but opens other issues related to the choice of suitable algorithm for selecting features and subsequent classifier which were shown to be not unique [102] The use of more complex approaches relying on deep learning (especially convolutional neural networks) may alleviate most of the difficulties raised by handcrafted features even if other challenges arise like the number of data used for training and tuning hyper-parameters of the model. A very good overview of the available technique that may be potentially clinically efficient in a near future can be found in [103].

**Figure 5:**
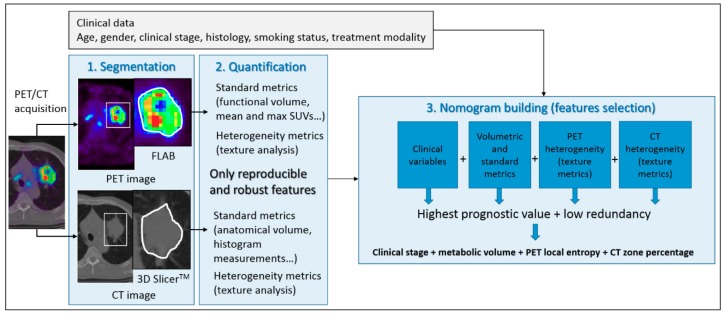
Workflow of a nomogram construction combining tumor and heterogeneity features extracted from both PET and CT components of routinely acquired FDG-PET scans in non-small cell lung cancers, allowing for better stratification among patients with stage II–III, compared to stage I. This research was originally published in EJNMMI [104]. © Springer.

Finally, a few studies have investigated the potential combination of image-derived features from multimodal imaging or associated to clinical data [95,104,105,106]. One example of a nomogram construction, published by Desseroit et al. [104], combining tumor and heterogeneity features extracted from both PET and CT components of routinely acquired FDG-PET scans in non-small cell lung cancers is shown in Figure 5. Reports in patients with mantle cell lymphoma also successfully applied this approach [107,108,109]. In this pathology, as demonstrated in a prospective study [107,108] and confirmed in a recent work by Mayerhoefer et al. [109], the combination of radiomic features with bio-clinical scores may possibly improve risk stratification. All these approaches form the basis for future works investigating the value of textural features in PET imaging and combining these methods will only reinforce the validity of the studies. In this regard, a recent study focused on this topic and successfully applied some of these approaches. In this work [95], Lucia et al. validated in two independent external cohorts of patients previously developed textural features-based models [110] relying on FDG PET and MRI for prediction of disease-free survival and locoregional control in locally advanced cervical cancer. Moreover, to adjust for the multicenter effects, they used the ComBat method, derived from genomic data analysis. They were able to identify two radiomics features indeed associated with worse outcome, confirming that more heterogeneous tumors have a poorer prognosis [95].

Radiomic is a promising field. Unlike histological biomarkers derived from invasive biopsy which sample only a small limited tumor region, as described above, radiomics non-invasively interrogate the whole tumor. Visualization of tumor heterogeneity is essential in the assessment of tumor aggressiveness and prognosis. Radiomic has an exceptional potential and may prove critical toward personalized medicine [83].

## 5. Conclusions

Nuclear medicine is one of the most dynamic medical fields. Advances in cancer biology knowledge together with the rise of new imaging techniques (new detection system and progress in imaging analysis) make this discipline a domain of tremendous and growing evolution. This dynamism is a real asset as personalized medicine has never been so relevant today. Indeed, PET imaging appears as an essential tool for non-invasive exploration of intratumoral and interlesional heterogeneity through the exploration of the distribution and uptake of a tracer or by using multiple radiopharmaceuticals, each providing different information. There is convincing evidence that the integration of PET imaging “profiling,” combining these approaches, associated to clinical, biological, or genomic data could improve tumor characterization and prognosis prediction to allow adequate patients stratification to therapeutic regimens. By combining at least one metabolic tracer with a phenotypic one, and by quoting Mankoff et Dehdashti [31], “it may then be possible to show that when it comes to molecular imaging, 1 plus 1 is greater than 2.”

## Figures and Tables

**Table 1:** Main validated positron-emission-tomography (PET) tracers and their principal indications (based on [10,11]).

Tracer	Metabolic Process	Principal Oncological Indications
11C-Methionine	Amino acid transport and protein synthesis	Diagnosis and grading of brain tumors
18F-Choline (FCH)	Phosphatidylcholine metabolism and cellular membrane turnover	Biopsy guidance of prostate cancer recurrence/primary staging in high-risk prostate cancer before surgical procedures or planning external beam radiation
18F-Fluoro-Deoxyglucose (FDG)	Glucose metabolism	Diagnosis/restaging of lung cancer, colorectal cancer, breast cancer, lymphoma, sarcoma, melanoma, head and neck cancer
18F-DOPA	Dopamine uptake and metabolism	Diagnosis of neuroendocrine tumors (NET)/documented NET metastasis in unknown primary
68Ga-DOTA-Peptides	Somatostatin receptors	Identification of primary tumor in patients with documented NET metastasis/assessment of NET disease extent before treatment
18F-Fluoroestradiol (FES)	Estrogen receptor	Status of tumor lesions to determine need for endocrine therapy in breast cancer
18F-Fluorothymidine (FLT)	Cellular proliferation and	Differential diagnosis between benign and malignant lesions/lymphoma staging and therapeutic evaluation
18-Sodium Fluoride (NaF)	Bone metabolism	Detection of bone involvement in tumors with elevated risk of bone metastasis
68Ga-Prostate-Specific Membrane Antigen (PSMA)	PSMA expression	Localization of tumor tissue in recurrent prostate cancer

**Table 2:** Common imaging heterogeneity parameters. (Based on [61,91]).

Order	Matrix	Name of the Parameter	Description of the Parameter
**First Order**		SUVmax	SUV value of the maximum intensity voxel within a region of interest (ROI)
		SUVpeak	Average SUV within a small ROI (usually, a 1-cm^3^ spherical volume)
**Second Order**		SUVmean	Average measure of SUV within a defined ROI
		Metabolic tumor volume (MTV)	Volume of a defined ROI
		Total lesion glycolysis (TLG)	Product of SUVmean × MTV
	**Grey-Level Co-Occurrence Matrix (GLCM)**	Contrast	Local variations in the GLCM
		Correlation	Joint probability occurrence of the specified pixel pairs
		Entropy	Texture randomness or irregularity
		Energy	Sum of squared elements in the GLCM
		Homogeneity	Closeness of the distribution of elements to the diagonal
**High Order**	**Gray-Level Run-Length Matrix (GLRLM)**	Short run emphasis (SRE)	Distribution of short runs
		Long run emphasis (LRE)	Distribution of long runs
		High gray level run emphasis (HGRE)	Distribution of high grey level values runs
		Grey-level non-uniformity (GLNU)	Similarity of grey level values throughout the image
		Run percentage (RP)	Homogeneity and distribution of runs of an image in a specific direction
	**Gray-Level Zone Size Encoding Method (GLZSM)**	High gray-level zone emphasis (HGZE)	Distribution of high grey level values zones
		Zone length non uniformity (ZLNU)	Similarity of zone length throughout the image
		Zone percentage (ZP)	Homogeneity and distribution of zones of an image in a specific direction
		Short zone emphasis (SZE)	Distribution of small zones
	**Neighborhood Grey Tone Difference Matrix (NGTDM)**	Coarseness	Granularity within an image.

## References

[1] Tabassum D.P., Polyak K. (2015). Tumorigenesis: It takes a village. Nat. Rev. Cancer.

[2] Dagogo-Jack I., Shaw A.T. (2018). Tumour heterogeneity and resistance to cancer therapies. Nat. Rev. Clin. Oncol..

[3] Lambin P., Rios-Velazquez E., Leijenaar R., Carvalho S., Van Stiphout R.G., Granton P., Zegers C.M., Gillies R., Boellard R., Dekker A. (2012). Radiomics: Extracting more information from medical images using advanced feature analysis. Eur. J. Cancer.

[4] Aerts H.J.W.L., Velazquez E.R., Leijenaar R.T.H., Parmar C., Grossmann P., Cavalho S., Bussink J., Monshouwer R., Haibe-Kains B., Rietveld D. (2014). Decoding tumour phenotype by noninvasive imaging using a quantitative radiomics approach. Nat. Commun..

[5] Bensch F., Van Der Veen E.L., Hooge M.N.L.-D., Jorritsma-Smit A., Boellaard R., Kok I.C., Oosting S.F., Schröder C.P., Hiltermann T.J.N., Van Der Wekken A.J. (2018). 89Zr-atezolizumab imaging as a non-invasive approach to assess clinical response to PD-L1 blockade in cancer. Nat. Med..

[6] Mu C.-Y., Huang J.-A., Chen Y., Chen C., Zhang X.-G. (2011). High expression of PD-L1 in lung cancer may contribute to poor prognosis and tumor cells immune escape through suppressing tumor infiltrating dendritic cells maturation. Med. Oncol..

[7] Chen L., Han X. (2015). Anti–PD-1/PD-L1 therapy of human cancer: Past, present, and future. J. Clin. Investig..

[8] Krishnamurthy A., Jimeno A. (2017). Atezolizumab: A novel PD-L1 inhibitor in cancer therapy with a focus in bladder and non-small cell lung cancers. Drugs Today.

[9] Fehrenbacher L., Spira A., Ballinger M., Kowanetz M., Vansteenkiste J., Mazieres J., Park K., Smith D., Artal-Cortes A., Lewanski C. (2016). Atezolizumab versus docetaxel for patients with previously treated non-small-cell lung cancer (POPLAR): A multicentre, open-label, phase 2 randomised controlled trial. Lancet.

[10] Jadvar H., Delgado-Bolton R., Nadel H., Rohren E., Zukotynski K., Kauffman J., Ahuja S., Colletti P.M., Esposito G., Krause B.J. (2017). Appropriate Use Criteria for 18 F-FDG PET/CT in Restaging and Treatment Response Assessment of Malignant Disease. J. Nucl. Med..

[11] Lopci E., Nanni C., Castellucci P., Montini G.C., Allegri V., Rubello D., Chierichetti F., Ambrosini V., Fanti S. (2010). Imaging with non-FDG PET tracers: Outlook for current clinical applications. Insights Imaging.

[12] Chudgar A.V., Mankoff D.A. (2017). Molecular Imaging and Precision Medicine in Breast Cancer. PET Clin..

[13] Koleva-Kolarova R.G., Greuter M.J., Feenstra T.L., Vermeulen K.M., De Vries E.F., Parkin D., Buskens E., De Bock G.H. (2018). Molecular imaging with positron emission tomography and computed tomography (PET/CT) for selecting first-line targeted treatment in metastatic breast cancer: A cost-effectiveness study. Oncotarget.

[14] Kurland B.F., Peterson L.M., Lee J.H., Schubert E.K., Currin E.R., Link J.M., Krohn K.A., Mankoff D.A., Linden H.M. (2017). Estrogen receptor binding (FES PET) and glycolytic activity (FDG PET) predict progression-free survival on endocrine therapy in patients with ER+ breast cancer. Clin. Cancer Res..

[15] Mortimer J.E., Dehdashti F., Siegel B.A., Katzenellenbogen J.A., Fracasso P., Welch M.J. (1996). Positron emission tomography with 2-[18F]Fluoro-2-deoxy-D-glucose and 16alpha-[18F]fluoro-17beta-estradiol in breast cancer: Correlation with estrogen receptor status and response to systemic therapy. Clin. Cancer Res..

[16] Liao G.J., Clark A.S., Schubert E.K., Mankoff D.A. (2016). 18F-Fluoroestradiol PET: Current Status and Potential Future Clinical Applications. J. Nucl. Med..

[17] Van Kruchten M., Glaudemans A.W.J.M., De Vries E.F.J., Beets-Tan R.G.H., Schröder C.P., Dierckx R.A., De Vries E.G.E., Hospers G.A.P. (2012). PET Imaging of Estrogen Receptors as a Diagnostic Tool for Breast Cancer Patients Presenting with a Clinical Dilemma. J. Nucl. Med..

[18] Dijkers E.C., Kosterink J.G., Rademaker A.P., Perk L.R., Van Dongen G.A., Bart J., De Jong J.R., De Vries E.G., Hooge M.N.L.-D. (2009). Development and Characterization of Clinical-Grade 89Zr-Trastuzumab for HER2/neu ImmunoPET Imaging. J. Nucl. Med..

[19] Baum R.P., Prasad V., Schuchardt C., Orlova A., Wennborg A., Tolmachev V., Feldwisch J., Müller D. (2010). Molecular Imaging of HER2-Expressing Malignant Tumors in Breast Cancer Patients Using Synthetic 111In- or 68Ga-Labeled Affibody Molecules. J. Nucl. Med..

[20] Tamura K., Kurihara H., Yonemori K., Tsuda H., Suzuki J., Kono Y., Honda N., Kodaira M., Yamamoto H., Yunokawa M. (2013). 64Cu-DOTA-Trastuzumab PET Imaging in Patients with HER2-Positive Breast Cancer. J. Nucl. Med..

[21] Jauw Y.W.S., Oordt C.W.M.-V.D.H.V., Hoekstra O.S., Hendrikse N.H., Vugts D.J., Zijlstra J.M., Huisman M.C., Van Dongen G.A.M.S. (2016). Immuno-Positron Emission Tomography with Zirconium-89-Labeled Monoclonal Antibodies in Oncology: What Can We Learn from Initial Clinical Trials?. Front. Pharmacol..

[22] Gebhart G., Lamberts L.E., Wimana Z., Garcia C., Emonts P., Ameye L., Stroobants S., Huizing M., Aftimos P., Tol J. (2016). Molecular imaging as a tool to investigate heterogeneity of advanced HER2-positive breast cancer and to predict patient outcome under trastuzumab emtansine (T-DM1): The ZEPHIR trial. Ann. Oncol..

[23] Clark A.S., DeMichele A., Mankoff D. (2016). HER2 imaging in the ZEPHIR study. Ann. Oncol..

[24] Maguire L.H., Thomas A.R., Goldstein A.M. (2015). Tumors of the neural crest: Common themes in development and cancer: Tumors of the Neural Crest. Dev. Dyn..

[25] Bar-Sever Z., Biassoni L., Shulkin B., Kong G., Hofman M.S., Lopci E., Manea I., Koziorowski J., Castellani R., Boubaker A. (2018). Guidelines on nuclear medicine imaging in neuroblastoma. Eur. J. Nucl. Med. Mol. Imaging.

[26] Kennedy A.S., Dezarn W.A., McNeillie P., Coldwell D., Nutting C., Carter D., Murthy R., Rose S., Warner R.R.P., Liu D. (2008). Radioembolization for Unresectable Neuroendocrine Hepatic Metastases Using Resin 90Y-Microspheres: Early Results in 148 Patients. Am. J. Clin. Oncol..

[27] Liu Y.-L., Lu M.-Y., Chang H.-H., Lu C.-C., Lin D.-T., Jou S.-T., Yang Y.-L., Lee Y.-L., Huang S.-F., Jeng Y.-M. (2016). Diagnostic FDG and FDOPA positron emission tomography scans distinguish the genomic type and treatment outcome of neuroblastoma. Oncotarget.

[28] Chan D.L., Pavlakis N., Schembri G.P., Bernard E.J., Hsiao E., Hayes A., Barnes T., Diakos C., Khasraw M., Samra J. (2017). Dual Somatostatin Receptor/FDG PET/CT Imaging in Metastatic Neuroendocrine Tumours: Proposal for a Novel Grading Scheme with Prognostic Significance. Theranostics.

[29] Zhang P., Yu J., Li J., Shen L., Li N., Zhu H., Zhai S., Zhang Y., Yang Z., Lu M. (2018). Clinical and Prognostic Value of PET/CT Imaging with Combination of 68Ga-DOTATATE and 18F-FDG in Gastroenteropancreatic Neuroendocrine Neoplasms. Contrast Media Mol. Imaging.

[30] Cistaro A., Quartuccio N., Caobelli F., Piccardo A., Paratore R., Coppolino P., Sperandeo A., Arnone G., Ficola U. (2015). 124I-MIBG: A new promising positron-emitting radiopharmaceutical for the evaluation of neuroblastoma. Nucl. Med. Rev..

[31] Mankoff D.A., Dehdashti F. (2009). Imaging Tumor Phenotype: 1 Plus 1 Is More than 2. J. Nucl. Med..

[32] Waseem N., Aparici C.M., Kunz P.L., Waseem N.L. (2019). Evaluating the Role of Theranostics in Grade 3 Neuroendocrine Neoplasms. J. Nucl. Med..

[33] Barrington S.F., Mikhaeel N.G., Kostakoglu L., Meignan M., Hutchings M., Müeller S.P., Schwartz L.H., Zucca E., Fisher R.I., Trotman J. (2014). Role of imaging in the staging and response assessment of lymphoma: Consensus of the International Conference on Malignant Lymphomas Imaging Working Group. J. Clin. Oncol..

[34] Cheson B.D., Ansell S., Schwartz L., Gordon L.I., Advani R., Jacene H.A., Hoos A., Barrington S.F., Armand P. (2016). Refinement of the Lugano Classification lymphoma response criteria in the era of immunomodulatory therapy. Blood.

[35] Kong F.-L., Ford R.J., Yang D.J. Managing Lymphoma with Non-FDG Radiotracers: Current Clinical and Preclinical Applications. https://www.hindawi.com/journals/bmri/2013/626910/.

[36] Buck A.K., Bommer M., Stilgenbauer S., Juweid M., Glatting G., Schirrmeister H., Mattfeldt T., Tepsic D., Bunjes D., Mottaghy F.M. (2006). Molecular Imaging of Proliferation in Malignant Lymphoma. Cancer Res..

[37] Nuutinen J., Leskinen S., Lindholm P., Söderström K.-O., Någren K., Huhtala S., Minn H. (1998). Use of carbon-11 methionine positron emission tomography to assess malignancy grade and predict survival in patients with lymphomas. Eur. J. Nucl. Med..

[38] Minamimoto R., Fayad L., Advani R., Vose J., Macapinlac H., Meza J., Hankins J., Mottaghy F., Juweid M., Quon A. (2016). Diffuse Large B-Cell Lymphoma: Prospective Multicenter Comparison of Early Interim FLT PET/CT versus FDG PET/CT with IHP, EORTC, Deauville, and PERCIST Criteria for Early Therapeutic Monitoring. Radiology.

[39] Herrmann K., Buck A.K., Schuster T., Abbrederis K., Blümel C., Santi I., Rudelius M., Wester H.-J., Peschel C., Schwaiger M. (2014). Week one FLT-PET response predicts complete remission to R-CHOP and survival in DLBCL. Oncotarget.

[40] Chantepie S., Hovhannisyan N., Guillouet S., Pelage J.-P., Ibazizene M., Bodet-Milin C., Carlier T., Gac A.-C., Reboursière E., Vilque J.-P. (2018). 18F-Fludarabine PET for Lymphoma Imaging: First-in-Humans Study on DLBCL and CLL Patients. J. Nucl. Med..

[41] Gourni E., Demmer O., Schottelius M., D’Alessandria C., Schulz S., Dijkgraaf I., Schumacher U., Schwaiger M., Kessler H., Wester H.-J. (2011). PET of CXCR4 Expression by a 68Ga-Labeled Highly Specific Targeted Contrast Agent. J. Nucl. Med..

[42] Herrmann K., Schottelius M., Lapa C., Osl T., Poschenrieder A., Hänscheid H., Lückerath K., Schreder M., Bluemel C., Knott M. (2016). First-in-Human Experience of CXCR4-Directed Endoradiotherapy with 177Lu- and 90Y-Labeled Pentixather in Advanced-Stage Multiple Myeloma with Extensive Intra- and Extramedullary Disease. J. Nucl. Med..

[43] Hindié E. (2017). The NETPET Score: Combining FDG and Somatostatin Receptor Imaging for Optimal Management of Patients with Metastatic Well-Differentiated Neuroendocrine Tumors. Theranostics.

[44] Gains J.E., Sebire N.J., Moroz V., Wheatley K., Gaze M.N. (2018). Immunohistochemical evaluation of molecular radiotherapy target expression in neuroblastoma tissue. Eur. J. Nucl. Med. Mol. Imaging.

[45] Gains J.E., Bomanji J.B., Fersht N.L., Sullivan T., D’Souza D., Sullivan K.P., Aldridge M., Waddington W., Gaze M.N. (2011). 177Lu-DOTATATE Molecular Radiotherapy for Childhood Neuroblastoma. J. Nucl. Med..

[46] Kayano D., Kinuya S. (2015). Iodine-131 Metaiodobenzylguanidine Therapy for Neuroblastoma: Reports So Far and Future Perspective. Sci. World J..

[47] Deubzer H., Hundsdoerfer P., Fuchs J., Prasad V., Timmermann B., Astrahantseff K., Berthold F., Simon T., Hero B., Schulte J.H. (2017). 2017 GPOH Guidelines for Diagnosis and Treatment of Patients with Neuroblastic Tumors. Klin. Pädiatr..

[48] Kong G., Hofman M.S., Murray W.K., Wilson S., Wood P., Downie P., Super L., Hogg A., Eu P., Hicks R.J. (2016). Initial Experience with Gallium-68 DOTA-Octreotate PET/CT and Peptide Receptor Radionuclide Therapy for Pediatric Patients with Refractory Metastatic Neuroblastoma. J. Pediatr. Hematol..

[49] Strosberg J., El-Haddad G., Wolin E., Hendifar A., Yao J., Chasen B., Mittra E., Kunz P.L., Kulke M.H., Jacene H. (2017). Phase 3 Trial of 177Lu-Dotatate for Midgut Neuroendocrine Tumors. N. Engl. J. Med..

[50] Navalkissoor S., Flux G., Bomanji J. (2017). Molecular radiotheranostics for neuroendocrine tumours. Clin. Med..

[51] Lee S.T., Kulkarni H.R., Singh A., Baum R.P. (2017). Theranostics of Neuroendocrine Tumors. Visc. Med..

[52] Bailly C., Cléry P.-F., Faivre-Chauvet A., Bourgeois M., Guérard F., Haddad F., Barbet J., Chérel M., Kraeber-Bodéré F., Carlier T. (2016). Immuno-PET for Clinical Theranostic Approaches. Int. J. Mol. Sci..

[53] Giesen D., Jalving M., Moek K.L., Kok I.C., De Groot D.J.A., Fehrmann R.S., Hooge M.N.L.-D., Brouwers A.H., De Vries E.G. (2017). Theranostics Using Antibodies and Antibody-Related Therapeutics. J. Nucl. Med..

[54] Lamberts L.E., Williams S.P., Van Scheltinga A.G.T., Hooge M.N.L.-D., Schroder C.P., Gietema J.A., Brouwers A.H., De Vries E.G. (2015). Antibody Positron Emission Tomography Imaging in Anticancer Drug Development. J. Clin. Oncol..

[55] Kraeber-Bodere F., Bailly C., Chérel M., Chatal J.-F. (2016). ImmunoPET to help stratify patients for targeted therapies and to improve drug development. Eur. J. Nucl. Med. Mol. Imaging.

[56] Sörensen J., Velikyan I., Sandberg D., Wennborg A., Feldwisch J., Tolmachev V., Orlova A., Sandström M., Lubberink M., Olofsson H. (2016). Measuring HER2-Receptor Expression In Metastatic Breast Cancer Using [68Ga]ABY-025 Affibody PET/CT. Theranostics.

[57] Fletcher J.W., Djulbegovic B., Soares H.P., Siegel B.A., Lowe V.J., Lyman G.H., Coleman R.E., Wahl R., Paschold J.C., Avril N. (2008). Recommendations on the Use of 18F-FDG PET in Oncology. J. Nucl. Med..

[58] Czernin J., Allen-Auerbach M., Nathanson D., Herrmann K. (2013). PET/CT in Oncology: Current Status and Perspectives. Curr. Radiol. Rep..

[59] Petersen H., Holdgaard P.C., Madsen P.H., Knudsen L.M., Gad D., Gravergaard A.E., Rohde M., Godballe C., Engelmann B.E., Bech K. (2016). FDG PET/CT in cancer: Comparison of actual use with literature-based recommendations. Eur. J. Nucl. Med. Mol. Imaging.

[60] Thie J.A. (2004). Understanding the standardized uptake value, its methods, and implications for usage. J. Nucl. Med..

[61] Carlier T., Bailly C. (2015). State-Of-The-Art and Recent Advances in Quantification for Therapeutic Follow-Up in Oncology Using PET. Front. Med..

[62] Keyes J.W. (1995). SUV: Standard uptake or silly useless value?. J. Nucl. Med..

[63] Okada J., Oonishi H., Yoshikawa K., Itami J., Uno K., Imaseki K., Arimizu N. (1994). FDG-PET for predicting the prognosis of malignant lymphoma. Ann. Nucl. Med..

[64] Schöder H., Noy A., Gönen M., Weng L., Green D., Erdi Y.E., Larson S.M., Yeung H.W.D. (2005). Intensity of 18fluorodeoxyglucose uptake in positron emission tomography distinguishes between indolent and aggressive non-Hodgkin’s lymphoma. J. Clin. Oncol..

[65] Bodet-Milin C., Kraeber-Bodéré F., Moreau P., Campion L., Dupas B., Le Gouill S., Drouet M., Delaunay C., Grenier N., Garrigou P. (2008). Investigation of FDG-PET/CT imaging to guide biopsies in the detection of histological transformation of indolent lymphoma. Haematologica.

[66] Bodet-Milin C., Bailly C., Meignan M., Beriollo-Riedinger A., Casasnovas R.-O., Devillers A., Lamy T., Santiago-Ribeiro M., Gyan E., Gallazzini-Crépin C. (2015). Predictive Power of FDG-PET Parameters at Diagnosis and after Induction in Patients with Mantle Cell Lymphoma, Interim Results from the LyMa-PET Project, Conducted on Behalf of the Lysa Group. Blood.

[67] Bodet-Milin C., Touzeau C., Leux C., Sahin M., Moreau A., Maisonneuve H., Morineau N., Jardel H., Gallazini-Crépin C., Gries P. (2010). Prognostic impact of 18F-fluoro-deoxyglucose positron emission tomography in untreated mantle cell lymphoma: A retrospective study from the GOELAMS group. Eur. J. Nucl. Med. Mol. Imaging.

[68] Bailly C., Carlier T., Touzeau C., Arlicot N., Kraeber-Bodéré F., Le Gouill S., Bodet-Milin C. (2019). Interest of FDG-PET in the Management of Mantle Cell Lymphoma. Front. Med..

[69] Bailly C., Carlier T., Berriolo-Riedinger A., Casasnovas O., Gyan E., Meignan M., Moreau A., Burroni B., Djaileb L., Gressin R. (2019). Prognostic value of FDG-PET in patients with mantle cell lymphoma: Results from the LyMa-PET Project. Haematologica.

[70] Eisenhauer E., Therasse P., Bogaerts J., Schwartz L., Sargent D., Ford R., Dancey J., Arbuck S., Gwyther S., Mooney M. (2009). New response evaluation criteria in solid tumours: Revised RECIST guideline (Version 1.1). Eur. J. Cancer.

[71] Therasse P., Arbuck S.G., Eisenhauer E.A., Wanders J., Kaplan R.S., Rubinstein L., Verweij J., Van Glabbeke M., Van Oosterom A.T., Christian M.C. (2000). New Guidelines to Evaluate the Response to Treatment in Solid Tumors. J. Natl. Cancer Inst..

[72] Schwartz L.H., Litiere S., De Vries E., Ford R., Gwyther S., Mandrekar S., Shankar L., Bogaerts J., Chen A., Dancey J. (2016). RECIST 1.1—Update and Clarification: From the RECIST Committee. Eur. J. Cancer.

[73] Wahl R.L., Jacene H., Kasamon Y., Lodge M.A. (2009). From RECIST to PERCIST: Evolving Considerations for PET Response Criteria in Solid Tumors. J. Nucl. Med..

[74] Al-Hajj M., Becker M.W., Wicha M., Weissman I., Clarke M.F. (2004). Therapeutic implications of cancer stem cells. Curr. Opin. Genet. Dev..

[75] Huff C.A., Matsui W., Smith B.D., Jones R.J. (2006). The paradox of response and survival in cancer therapeutics. Blood.

[76] Lin C., Itti E., Haioun C., Petegnief Y., Luciani A., Dupuis J., Paone G., Talbot J.-N., Rahmouni A., Meignan M. (2007). Early 18F-FDG PET for Prediction of Prognosis in Patients with Diffuse Large B-Cell Lymphoma: SUV-Based Assessment versus Visual Analysis. J. Nucl. Med..

[77] Wahl R.L., Zasadny K., Helvie M., Hutchins G.D., Weber B., Cody R. (1993). Metabolic monitoring of breast cancer chemohormonotherapy using positron emission tomography: Initial evaluation. J. Clin. Oncol..

[78] Bailly C., Carlier T., Jamet B., Eugene T., Touzeau C., Attal M., Hulin C., Facon T., Leleu X., Perrot A. (2018). Interim PET Analysis in First-Line Therapy of Multiple Myeloma: Prognostic Value of ΔSUVmax in the FDG-Avid Patients of the IMAJEM Study. Clin. Cancer Res..

[79] Matsui W., Huff C.A., Wang Q., Malehorn M.T., Barber J., Tanhehco Y., Smith B.D., Civin C.I., Jones R.J. (2004). Characterization of clonogenic multiple myeloma cells. Blood.

[80] Rasche L., Chavan S.S., Stephens O.W., Patel P.H., Tytarenko R., Ashby C., Bauer M., Stein C., Deshpande S., Wardell C. (2017). Spatial genomic heterogeneity in multiple myeloma revealed by multi-region sequencing. Nat. Commun..

[81] Rasche L., Kortüm K.M., Raab M.S., Weinhold N. (2019). The Impact of Tumor Heterogeneity on Diagnostics and Novel Therapeutic Strategies in Multiple Myeloma. Int. J. Mol. Sci..

[82] Pugachev A., Ruan S., Carlin S., Larson S.M., Campa J., Ling C.C., Humm J.L. (2005). Dependence of FDG uptake on tumor microenvironment. Int. J. Radiat. Oncol..

[83] Gillies R.J., Kinahan P.E., Hricak H. (2016). Radiomics: Images Are More than Pictures, They Are Data. Radiology.

[84] Lambin P., Leijenaar R.T., Deist T.M., Peerlings J., De Jong E.E., Van Timmeren J., Sanduleanu S., LaRue R.T., Even A.J., Jochems A. (2017). Radiomics: The bridge between medical imaging and personalized medicine. Nat. Rev. Clin. Oncol..

[85] Hatt M., Tixier F., Visvikis D., Cheze Le Rest C. (2017). Radiomics in PET/CT: More Than Meets the Eye?. J. Nucl. Med..

[86] Hatt M., Tixier F., Pierce L., Kinahan P.E., Rest C.C.L., Visvikis D. (2016). Characterization of PET/CT images using texture analysis: The past, the present … any future?. Eur. J. Nucl. Med. Mol. Imaging.

[87] Vallières M., Zwanenburg A., Badic B., Cheze Le Rest C., Visvikis D., Hatt M. (2018). Responsible Radiomics Research for Faster Clinical Translation. J. Nucl. Med..

[88] Berthon B., Spezi E., Galavis P., Shepherd T., Apte A., Hatt M., Fayad H., De Bernardi E., Soffientini C.D., Schmidtlein C.R. (2017). Toward a standard for the evaluation of PET-Auto-Segmentation methods following the recommendations of AAPM task group No. 211: Requirements and implementation. Med Phys..

[89] Zwanenburg A., Leger S., Vallières M., Löck S. (2016). Image biomarker standardisation initiative. arXiv.

[90] Tixier F., Le Rest C.C., Hatt M., Albarghach N.M., Pradier O., Metges J.-P., Corcos L., Visvikis D. (2011). Intratumor heterogeneity characterized by textural features on baseline 18F-FDG PET images predicts response to concomitant radiochemotherapy in esophageal cancer. J. Nucl. Med..

[91] Cook G.J.R., Siddique M., Taylor B.P., Yip C., Chicklore S., Goh V. (2014). Radiomics in PET: Principles and applications. Clin. Transl. Imaging.

[92] Lee J.W., Lee S.M. (2018). Radiomics in Oncological PET/CT: Clinical Applications. Nucl. Med. Mol. Imaging.

[93] Chalkidou A., O’Doherty M.J., Marsden P.K. (2015). False Discovery Rates in PET and CT Studies with Texture Features: A Systematic Review. PLoS ONE.

[94] Welch M.L., McIntosh C., Haibe-Kains B., Milosevic M.F., Wee L., Dekker A., Huang S.H., Purdie T.G., O’Sullivan B., Aerts H.J. (2019). Vulnerabilities of radiomic signature development: The need for safeguards. Radiother. Oncol..

[95] Lucia F., Visvikis D., Vallières M., Desseroit M.-C., Miranda O., Robin P., Bonaffini P.A., Alfieri J., Masson I., Mervoyer A. (2019). External validation of a combined PET and MRI radiomics model for prediction of recurrence in cervical cancer patients treated with chemoradiotherapy. Eur. J. Nucl. Med. Mol. Imaging.

[96] Wu J., Aguilera T., Shultz D., Gudur M., Rubin D.L., Loo B.W., Diehn M., Li R. (2016). Early-Stage Non–Small Cell Lung Cancer: Quantitative Imaging Characteristics of 18F Fluorodeoxyglucose PET/CT Allow Prediction of Distant Metastasis. Radiology.

[97] Carvalho S., Leijenaar R.T.H., Troost E.G.C., van Timmeren J.E., Oberije C., van Elmpt W., de Geus-Oei L.-F., Bussink J., Lambin P. (2018). 18F-fluorodeoxyglucose positron-emission tomography (FDG-PET)-Radiomics of metastatic lymph nodes and primary tumor in non-small cell lung cancer (NSCLC)—A prospective externally validated study. PLoS ONE.

[98] Ypsilantis P.-P., Siddique M., Sohn H.-M., Davies A., Cook G., Goh V., Montana G. (2015). Predicting Response to Neoadjuvant Chemotherapy with PET Imaging Using Convolutional Neural Networks. PLoS ONE.

[99] Arimura H., Soufi M., Kamezawa H., Ninomiya K., Yamada M. (2019). Radiomics with artificial intelligence for precision medicine in radiation therapy. J. Radiat. Res..

[100] Orlhac F., Boughdad S., Philippe C., Stalla-Bourdillon H., Nioche C., Champion L., Soussan M., Frouin F., Frouin V., Buvat I. (2018). A Postreconstruction Harmonization Method for Multicenter Radiomic Studies in PET. J. Nucl. Med..

[101] Chatterjee A., Vallieres M., Dohan A., Levesque I.R., Ueno Y., Saif S., Reinhold C., Seuntjens J. (2019). Creating Robust Predictive Radiomic Models for Data from Independent Institutions Using Normalization. IEEE Trans. Radiat. Plasma Med Sci..

[102] Upadhaya T., Vallieres M., Chatterjee A., Lucia F., Bonaffini P.A., Masson I., Mervoyer A., Reinhold C., Schick U., Seuntjens J. (2019). Comparison of Radiomics Models Built Through Machine Learning in a Multicentric Context with Independent Testing: Identical Data, Similar Algorithms, Different Methodologies. IEEE Trans. Radiat. Plasma Med Sci..

[103] Liu L., Chen J., Fieguth P., Zhao G., Chellappa R., Pietikäinen M. (2019). From BoW to CNN: Two Decades of Texture Representation for Texture Classification. Int. J. Comput. Vis..

[104] Desseroit M.-C., Visvikis D., Tixier F., Majdoub M., Guillevin R., Perdrisot R., Le Rest C.C., Hatt M. (2016). Development of a nomogram combining clinical staging with (18)F-FDG PET/CT image features in non-small-cell lung cancer stage I-III. Eur. J. Nucl. Med. Mol. Imaging.

[105] Chan S.-C., Cheng N.-M., Hsieh C.-H., Ng S.-H., Lin C.-Y., Yen T.-C., Hsu C.-L., Wan H.-M., Liao C.-T., Chang K.-P. (2017). Multiparametric imaging using 18F-FDG PET/CT heterogeneity parameters and functional MRI techniques: Prognostic significance in patients with primary advanced oropharyngeal or hypopharyngeal squamous cell carcinoma treated with chemoradiotherapy. Oncotarget.

[106] Vallières M., Freeman C.R., Skamene S.R., El Naqa I. (2015). A radiomics model from joint FDG-PET and MRI texture features for the prediction of lung metastases in soft-tissue sarcomas of the extremities. Phys. Med. Boil..

[107] Bodet-Milin C., Bailly C., Meignan M., Berriolo-riedinger A., Devillers A., Hermine O., Carlier T., Hatt M., Kraeber-Bodéré F., Gouill S.L. (2015). Prognosis value of quantitative indices derived from initial FDG PET/CT in untreated mantle cell lymphoma patients enrolled in the Lyma trial, a LYSA study. Preliminary results. J. Nucl. Med..

[108] Carlier T., Bailly C., Hatt M., Kraeber-Bodéré F., Visvikis D., Gouill S.L., Bodet-Milin C. (2015). Quantification of intratumor heterogeneity derived from baseline FDG PET/CT in untreated mantle cell lymphoma patients enrolled in a prospective phase III trial of the LYSA group: Preliminary results. J. Nucl. Med..

[109] Mayerhoefer M.E., Riedl C.C., Kumar A., Gibbs P., Weber M., Tal I., Schilksy J., Schöder H. (2019). Radiomic features of glucose metabolism enable prediction of outcome in mantle cell lymphoma. Eur. J. Nucl. Med. Mol. Imaging.

[110] Lucia F., Visvikis D., Desseroit M.-C., Miranda O., Malhaire J.-P., Robin P., Pradier O., Hatt M., Schick U. (2018). Prediction of outcome using pretreatment 18F-FDG PET/CT and MRI radiomics in locally advanced cervical cancer treated with chemoradiotherapy. Eur. J. Nucl. Med. Mol. Imaging.

